# The Twenty-Sixth Annual Meeting of the American Dental Association

**Published:** 1886-09

**Authors:** 


### THE TWENTY-SIXTH ANNUAL MEETING OF THE AMERICAN DENTAL ASSOCIATION.

The Niagara meeting can scarcely be called a very brilliant success. There was a fair attendance and some good papers were read, but the general interest was not what it should have been. Some of the sections were practically without a report, while in others, instead of an advance in thought and progress in science, there was the same dreary round of stale subjects, the same old familiar common-places from the old familiar sources, that have marked the meetings from time out of mind. To be sure, many of the old things were said in a very impressive way, and some of the old speeches were revamped and newly turned for the occasion, but there certainly was a most unaccountable lack of originality and token of primitive research for a meeting of the best men in dentistry. We have become habituated to the boast that dentistry is the most progressive of all the sciences, and that our rate of progress is astounding. We have just passed another anniversary, and what has been done to make good this vaunt ?

During the past year the various dental journals have published to the world some meritorious papers—essays that indicated a depth of research, a breadth of investigation that is creditable to us as a profession. But with two or three exceptions, what evidence of study was presented at Niagara? There should have been a plethora of original papers, so that it would have been impossible to read them all, except by title. In reality, there was a dearth of essays, and one almost sighed for another chapter of Stephen Pearl Andrews "Alwato" to break the monotony.

Sections I, II, III and IV presented little that was worthy of their subjects. Sections V, VI and VII did better, but even they did not rise to the full dignity of the occasion. There was less of windy rhetoric in the discussions than has sometimes been heard, but they were in the main spiritless. When meetings of the Sections were called it was with the extremest difficulty that some of them could get a quorum for the transaction of the necessary business. Members would not attend, and when they did had little to say. Meetings of important Sections were held with but three or four present, and the matter that was to be presented before the society was seldom properly digested. The officers of a Section cannot do the work of it alone, and unless they are properly sustained by the members we shall continue to see the same inertia manifested. When the secretary called the roll of members that they might choose the Section in which they would work during the coming year, there were but a few feeble responses, and many of those present were seen to slide toward the door.

One thing that interfered with the scientific success of the meeting was the miserable political maneuvering, the wire-pulling, and the factional spirit which was early manifested. Even on Monday, the day before the commencement of the session, the politicians were on hand and had begun their dirty work. There was a constant button-holing of members in every nook and corner, and all the resources of ward bummers were called in play to carry desired political ends. There were members who took no part in the discussions, or interest in the meeting aside from the trials of party strength which were afforded by the occasional votes. The attention of members was constantly distracted from the business in hand by the cabals of these politicians. Is it not strange that men will travel long distances and be at heavy expense to attend a meeting in which they have no interest aside from their desire to manipulate the political wires? To such a limitless extent was this contemptible business carried at Niagara, that many reputable members left before the close of the meeting, completely disgusted at the exhibition.

Nor was this confined to any one class or party. "Pipe-laying" was begun months ago, and men were solicited to support this or that one for office when the time came. In the name of all that is honorable, must this thing continue? Meeting ostensibly for scientific purposes, must the whole proceedings be prostituted to the ambitions of any man, or class of men? Do members go to these gatherings for no other purpose than to elect this or that man to an office? We protest against this debasement of an honorable society to dishonorable ends. Unless it be stopped the meetings of the American Dental Association will be unworthy the attendance of those who love their profession. It is to be hoped that this year has witnessed the last of this dishonorable business, and that we shall not again see such an exhibition as was made at Niagara.

We have spoken strongly on this subject because we have felt deeply, and deem it the duty of the professional journals to enter a protest that shall be heeded.

The financial exhibit was a remarkable one. But we believe we are not alone in the opinion that no organization like this has any right to thousands of dollars in its treasury. It is not supposed to be a money-making institution, and while the treasury should be in a healthy condition, surplus funds should be expended in the fostering of scientific study. The society did well in voting liberal sums this year to such of the Sections as are engaged in original investigation.

What can be done to make the society better? To our mind, unless the Section work be better done and a greater interest be manifested in them by the members, they should be abandoned, and a return made to the old committee method of work, which was successful for so many years.

The society should publish its own transactions, and not rest under obligations to any one man or firm. This is a delicate subject for us to approach, but we believe that we are entirely conscientious in our views. There is no questioning the manner in which the work has been done. The volumes of transactions published by the S. S. White Dental Manufacturing Co. are models of beauty and excellence, and that firm has had no selfish motive in doing it. They have had little to gain, while the expense has been considerable. But it is not a dignified position for a National Association to assume, when it places its records in the hands of any private firm whatever, and there is no disputing the fact that papers are withheld which, under a different method of publishing, would be presented to the society. If the present course is to be pursued, no one will question the fitness of giving the work to the present publishers. But it is a matter which, to our mind, should be done by the society itself.

There is another view of this method, which is that it should be the policy of the society rather to discourage than to countenance the exhibition of dental manufacturers and dealers at the meetings. A dental depot in the same building with the meeting constantly tends to distract attention from the papers and discussions. It is impossible to keep dentists out of the stock room, no matter how faithfully the proprietor may strive to do so. Members will gather in knots to discuss this or that appliance and to purchase goods, and their attention and interest is thus centered at the wrong place. We appeal to all those who have been in any way responsible for the conduct of the meetings, to say if the greatest difficulty they have encountered has not been to draw members from the depots to the hall when the time has come for opening the sessions. During the late meeting the President was not once able to open a session within twenty minutes of the hour, because of the impossibility to obtain a quorum. We hope that at the next meeting there will be no exhibition of dental goods allowed within the precincts of the building devoted to the sessions, and that to this end a hall will be engaged that has no rooms adjoining which can be used for such purposes. We do not mean to speak in detraction of the dental displays. On the contrary, it is because they are of such interest to the dentist that we think they should be kept at a distance. The American Dental Association does not meet for the purpose of affording members and others an opportunity to purchase stock.

There are other things concerning which we desire to speak, but the lack of space forbids, for the present. We do not wish to appear in the role of a universal fault-finder, but some one should give voice to a feeling that is widespread, and therefore we have spoken.

